# Prevalence and Associated Factors of Self-Treatment among the Elderly—A Comparative Study between Empty and Non-Empty Nesters in Shandong, China

**DOI:** 10.3390/ijerph17217880

**Published:** 2020-10-27

**Authors:** Zhaorong Gao, Lingzhong Xu, Wenzhe Qin, Jiao Zhang, Jinling Zhou, Fangfang Hu, Zhuang Hong

**Affiliations:** 1 School of Public Health, Cheeloo College of Medicine, Shandong University, Jinan 250012, China; gaozr20160104@163.com (Z.G.); qinwenzhe09@163.com (W.Q.); zhangjiao5263@163.com (J.Z.); hufang1218@yeah.net (F.H.); hongzhuang@mail.sdu.edu.cn (Z.H.); 2 NHC Key Laboratory of Health Economics and Policy Research, Cheeloo College of Medicine, Shandong University, Jinan 250012, China; 3 Shandong University Center for Health Economics Experiment and Public Policy Research, Shandong University, Jinan 250012, China; 4 School of Medicine and Health Management, Cheeloo College of Medicine, Shandong University, Jinan 250012, China; zhoujl@sdu.edu.cn

**Keywords:** self-treatment, associated factors, elderly, empty nesters, non-empty nesters

## Abstract

(1) Objectives: With an aging society in China, self-treatment now plays an important role in health care among older adults, but it can be problematic. This study aims to explore and compare the self-treatment behavior among empty and non-empty nesters. (2) Methods: Using a multi-stage stratified random cluster sampling method, a total of 4366 elderly people aged 60 and above from Shandong Province, China, were enrolled in this study. Data were collected through a structured questionnaire. Binary logistic regression was used to analyze the associated factors of self-treatment. (3) Results: The prevalence of self-treatment in empty nesters was significantly lower than that in non-empty ones (74.0% vs. 83.3%). Binary logistic regression analysis showed that higher educational level and poorer self-rated economic status were negatively associated with self-treatment in empty nesters, while unemployed and urban and rural residents’ basic medical insurance were positively associated with self-treatment in non-empty ones. (4) Conclusions: The study indicated that empty nesters had lower likelihood of self-treatment than non-empty ones. Empty nesters with better socioeconomic status were more likely to use self-treatment; by contrast, non-empty nesters with relatively poorer socioeconomic status were more inclined to self-treatment. Targeted interventions should be developed to maximize the effectiveness of self-treatment and reduce health risks.

## 1. Introduction

The number of older population over 60 years was 250 million at the end of 2018, accounting for 17.9% of the total population in China. People above the age of 65 are projected to represent 26% of China’s population by 2050 according to a report by the World Bank [1,2]. China is inevitably under the pressure of rapid aging. A major feature of China’s population aging is the transition of elderly household patterns. Living with children is the typical Chinese traditional family pattern for older people [3]. But due to many factors, such as the one-child parents entering the elderly, the imbalance of economic development, the acceleration of urbanization, and population migration, the proportion of elders living alone or living with their spouse continues to rise. These elders are called “empty nesters” [3,4,5,6]. The estimated number of empty nester households will account for 90% of the elderly households by 2030 [7]. Due to the lack of affection and spiritual comfort from their children for a long period, the empty nest elderly might have a worse health status. With the increasing number of empty nest elderly, the demand on health services for the elderly has become a major challenge facing us.

When ill, individuals can seek professional health care, self-treat, or do nothing [8]. Self-treatment refers to treating oneself without professional help to alleviate an illness or a condition [9], including self-medication or massage, hot compress or other auxiliary therapy. The definition of self-treatment differs across different publications [8,10,11]. In developing countries, self-treatment is increasing rapidly. A systematic review showed that the prevalence of self-medication among the elderly varies from 20% to 60% in most studies [12]. An Iranian study indicated that 80% of patients chose self-treatment [11]. In China, the fourth National Health Service Survey released that 38.0% of patients did not go to see a doctor, of which 70.0% used self-treatment [13]. The data of the China Health and Retirement Longitudinal Study (CHARLS) in 2013 showed that the prevalence of self-treatment among the middle-aged and elderly patients with non-communicable chronic diseases was 64.04% [14]. Compared with other professional hospital-based healthcare approaches (inpatient and outpatient treatments), correct self-treatment can be helpful for both individuals and medical health system, such as convenience, accessibility, lower medical costs, complementing the health care system, and relieving the burden of limited medical services. It can effectively alleviate the problems of difficulty and expense when seeing a doctor [8,15], and appropriate self-treatment can also avoid some unnecessary waste of medical resources [16]. But the negative effects of incorrect self-treatment cannot be ignored either. Irrational self-treatment, such as false self-diagnosis, drug abuse, missing optimal treatment, side effects or interaction with other drugs, can cause serious clinical consequences and unnecessary economic loss [15,17].

Older adults may be more likely to choose self-treatment due to the overall worse health status [18,19]. Previous studies have examined the potential determinates of self-treatment among the elderly. The decision to choose self-treatment involves a complex mechanism and is influenced by various factors, including individual, household, accessibility and medical insurance system factors [10,20]. Few studies, however, exist to date in China focusing on the roles of social changes and living arrangements in the use of self-treatment. Empty nesters may have a higher prevalence of self-treatment due to low socioeconomic status. Consequently, there is a clear need to evaluate nest status in the intensity of using self-treatment.

To extend the literature, the aims of this study are to identify the difference between empty and non-empty nesters in self-treatment behavior and comparatively evaluate the risk factors, respectively. This aims to provide a reference for formulating targeted interventions to improve self-treatment effectively.

## 2. Materials and Methods 

### 2.1. Data and Sample

Our data were collected from the Survey of Shandong Elderly Family Health Service, which was conducted by Shandong University in 2017. This study was approved by the Academic Research Ethics Committee of Shandong University. We employed a stratified multi-stage random sampling method to select Weihai, Weifang, and Heze as survey locations based on the gross domestic product (high, media, low) and geographical location (east, middle, and west). After verbal informed consent was obtained from interviewees, 7088 elderly people aged 60 years or older were interviewed individually using a structured questionnaire. Of these, 18 did not complete the survey. In total, 7070 individuals were included in the final sample. The detailed sampling methods have been described in a previously published article [21].

The current study focused on respondents who reported the presence of illness during the two-week period before they were interviewed with a sample of *n* = 4366.

### 2.2. Dependent Variable

In this article, self-treatment refers to “the scenario where a person takes medication (including over-the counter drugs or prescribed drug already in family) or other approaches, such as massage, hot compress or other auxiliary therapy, to cope with illness conditions without professional help” [9]. It was measured by a two-category qualitative question “Have you ever self-treatment during the two weeks?”

### 2.3. Independent Variables

Based on the location of residence of their adult children, we divided participants into two groups: empty nesters (living with their spouse, and their adult children had lived away from their village/community at least 6 months, or living alone, albeit with one or more adult children in the same village/community) and non-empty nesters (living with children, including those who live with one or more adult children).

Data on the demographic, socioeconomic, and health-related variables were collected using a self-designed questionnaire, including gender (male, female), age (60–69, 70–79, ≥ 80), education (no schooling, primary school, junior school and above), employment (employed, retired, unemployed), marital status (single, couple), insurance (Urban and Rural Residents Basic Medical Insurance (URRBMI), Urban Employee Basic Medical Insurance (UEBMI), others, or none), self-rated economic status (wealthy, not wealthy but not worried about livelihood, not wealthy and worried about livelihood, or poor), self-rated health status (good, medium, bad), and chronic diseases (yes, no).

### 2.4. Statistical Analysis

All statistical analyses were performed using SPSS 24.0 (IBM Crop, Armonk, NY, USA). A chi-square test was used to compare the differences in basic characteristics between empty and non-empty nesters. A univariate logistic regression model and multivariate logistic regression model were used to explore the factors among empty and non-empty nest elderly respectively. Three binary logistic regression models were used to identify the difference in self-treatment between empty nesters and non-empty nesters. Model 1: univariate model. Model 2: binary logistic regression model, adjusted covariates for social-economic factors. Model 3: In addition to the factors in Model 2, health-related factors were included. Furthermore, a secondary analysis were performed to compare the differences in self-treatment among the 3 subtypes of”empty nesters”. Association were determined by odds ratios (ORs) and 95% confidence intervals (CIs). *p*-Values < 0.05 were considered to be statistically significant.

## 3. Results

### 3.1. Basic Characteristics

Table 1 presents the basic information of subjects. The study included 4366 seniors with an average age of 70.01 years (standard deviation (SD) = 6.25), of which 36.6% were men and 63.4% were women. 1592 (36.5%) were empty nesters and 2774 (63.5%) were non-empty ones. Most of them (74.8%) had a qualification of primary school and below, more than 90% had one or more type of chronic diseases. Regarding self-treatment characteristics, the result showed that 3490 (79.9%) elderly had self-treatment in two weeks and 876 (20.1%) had not. Among these 3490 elderly who had self-treatment, 1178 (33.8%) were empty nesters and 2312 (66.2%) were non-empty ones. Non-empty nesters were more inclined to use self-treatment (83.3% vs. 74.0%, *p* < 0.001) than empty nesters.

### 3.2. Associations with Self-Treatment in Different Living Arrangements

Table 2 reports the association of self-treatment and living arrangement. After adjusting covariates for social-economic factors and health related factors, model 3 showed that non-empty nesters were more likely to use self-treatment than the empty ones. (OR = 1.749, 95% CI = 1.496–2.045, *p* < 0.001). All of the three models indicated the difference in self-treatment among empty and non-empty nesters. In addition, there were no significant differences in self-treatment have been seen between the three subgroups of the empty nesters.

### 3.3. Associated Factors of Self-Treatment among Empty Nesters

Table 3 shows the factors associated with self-treatment behavior among empty nesters. Univariate analysis indicated that age, education, self-rated economic status, self-rated health status, and chronic diseases were significantly associated with self-treatment. Multivariate logistic analysis showed that poorer economic status (*p* = 0.041) and chronic diseases (*p* = 0.029) were positively associated with self-treatment. A higher education level (*p* = 0.001) was negatively associated with self-treatment.

### 3.4. Associated Factors of Self-Treatment among Non-Empty Nesters

As shown in Table 4, univariate analysis indicated that employment, insurance and chronic diseases were related to self-treatment in non-empty nesters. Multivariate logistic analysis showed that being unemployed (*p* = 0.001), and chronic diseases (*p* < 0.001) were increased the likelihood of self-treatment. Elderly with other types of insurance (*p* = 0.015) were less likely to use self-treatment.

## 4. Discussion

The study showed that the prevalence of self-treatment among the elderly who reported the presence of illness during the two-week period before they were interviewed in Shandong, China, was 79.9%, which was higher than other similar studies [19,22]. It is difficult to compare with other studies due to the use of different definitions of self-treatment in each study. With the development of the economy and society, the access channels for health information is becoming more and more diversified, which plays a certain role in promoting self-treatment behavior.

Our results showed that the prevalence of self-treatment in empty nesters was lower than that in non-empty ones (74.0% vs. 83.3%), and the difference was significant after adjusting variables for social-economic and health-related factors. It is inconsistent with our hypothesis that the empty nesters may have a higher prevalence of self-treatment due to the poorer socioeconomic status. The possible explanation may be that China has improved the accessibility and equality for health services effectively in the past few years. A previous study [3] conducted in Shandong, China in 2011 showed that the empty nesters have a higher non-use rate of healthcare services than non-empty ones due to the fact that the higher demand for health services cannot be met equally, but another study conducted in Sinan county, China, in 2018 was inconsistent with it, which found that the empty nesters were less likely to choose self-treatment. After the ‘healthy China 2030’ plan released by the central government in 2016, a series of health poverty alleviation projects were implemented with the goal of strengthening financial risk protection against illness for the financially backward population [23,24,25]. A study indicated that more than 4.2 million poor patients with serious or chronic diseases have been treated due to the health poverty alleviation project [26]. Another important explanation for non-empty nesters having a higher prevalence of self-treatment than empty ones is that the family caregivers are often equipped with some knowledge and common skills of healthcare tasks due to the increased frailty of the elderly, and the non-empty nesters would usually deal with their discomfort with the support of their adult children [20].The results suggests that it is important to strengthen health education for elderly and family caregivers to make them understand the risks of improper self-treatment.

However, our separate analysis of empty and non-empty nesters showed that the effect of the socio-economic status on the behavior of self-treatment is significant for the empty nesters, but is not for the non-empty nesters. Among the empty nesters, the elderly who reported better economic status were more likely to use self-treatment. On the one hand, our view is that the targeted health poverty alleviation policy effectively improved the utilization of health services for the poorer population [27,28,29]. On the other hand, the possible explanation is that the elderly in poverty may have difficulty self-purchasing drugs with out-of-pocket money, which cannot be covered by medical insurance [20], and the elderly with higher affordability have more access to medical resources outside hospitals. In addition, education was negatively associated with self-treatment in empty nesters, educational level is related to the ability to access health information, elderly who with higher education usually have higher health literacy. However, even though our results showed a statistical significance between education and behavior, the clinical significance of the self-treatment rate of three groups of education level in Table 3, 77.9% in the least education and 70.8% in the most education, does not seem too impressive. We will further explore the relationship between education and self-treatment in the following research.

Regarding the non-empty nesters, there was no significant difference in using self-treatment among the elderly with different economic status. Except for the fact that empty nest elders are given more attention and social support than non-empty nesters due to their special physical and psychological needs, the health poverty alleviation project may be more significant in improving their health service utilization [30,31,32,33,34]. Furthermore, according to the need theory, the basic material needs must be fulfilled, and then additional economic resources may affect subjective behaviors with respect to non-material needs, but to a lesser degree [35,36]. This study used self-reported economic status, rather than objective income measures. It is subjective and can measure a person’s evaluation of their own socio-economic conditions more accurately that those less affected by the external factors. Compared with empty nesters, non-empty nesters have a higher demand for economic satisfaction and less additional economic resources to affect their medical behavior. However, insurance and employment were associated with self-treatment among non-empty nesters. URRBMI increased the likelihood of self-treatment compared with other types of commercial insurance. In addition, being unemployed was a predictor to self-treatment also. The findings suggest that after meeting basic material needs, relative income levels are negatively correlated with self-treatment among non-empty nesters.

Consistent with other similar studies [8,19,37,38], our results showed that chronic diseases was one common predictor of self-treatment among both empty and non-empty nesters. It should be noted that our study population include the elderly who had one or more chronic diseases but in a relatively stable status and need long-term medication, which may cause a higher prevalence of self-treatment among the elderly with chronic diseases.

There are some limitations in this study. First, this is a cross-sectional study and, therefore, the resulting factors associated with self-treatment behavior cannot determine causality. Second, the data were self-reported, and there might be a possible recall bias for most questionnaire data. Third, as for the definition of self-treatment, we only explained to the respondents before the survey that self-treatment refers to “the scenario where a person takes medication (including over-the counter drugs or prescribed drug already in family) or other approaches, such as massage, hot compress or other auxiliary therapy, to cope with illness conditions without professional help”, and it cannot be defined in detail. For example, it is not clear that whether it was self-treatment when they did exercises to relieve neck pain even though the exercises were recommended by a physical therapist. Therefore, this situation may cause potential additional biases. Finally, there may be more confounding factors than those available for consideration in this study. In addition, our study did not involve the self-treatment rate of single diseases, and the next step of our research can explore the diseases with the highest prevalence of self-treatment and find the priority management of diseases.

## 5. Conclusions

The prevalence of self-treatment among the elderly in Shandong, China, was 79.9%. Non-empty nest elderly had a higher prevalence of self-treatment than empty nesters. Our result showed that empty nesters with better socioeconomic status were more likely to use self-treatment, while non-empty nesters with relatively poorer socioeconomic status were more inclined to self-treatment. The findings from our study could provide the government with some information about the use of self-treatment among empty and non-empty nesters. Interventions targeting identified at-risk subgroups should be developed to maximize the effectiveness of self-treatment and reduce health risks.

## Figures and Tables

**Table 1:** Socio-demographic characteristics of the elderly in Shandong, China (*n* = 4366).

Characteristics	Total	Empty Nest	Non-Empty	χ^2^	*p*-Value
	*n* (%)	*n* (%)	*n* (%)		
*n*	4366 (100.0)	1592 (26.5)	2774 (63.5)		
Gender				1.001	0.317
Male	1597 (36.6)	567 (35.6)	1030 (37.1)		
Female	2769 (63.4)	1025 (64.4)	1744 (62.9)		
Age				9.890	0.007
60–69	2205 (50.5)	756 (47.5)	1449 (52.2)		
70–79	1805 (41.3)	691 (43.4)	1114 (40.2)		
≥80	356 (8.2)	145 (9.1)	211 (7.6)		
Education				3.193	0.203
No schooling	1480 (33.9)	521 (32.7)	959 (34.6)		
Primary school	1787 (40.9)	647 (40.6)	1140 (41.1)		
Junior school and above	1099 (25.2)	424 (26.6)	675 (24.3)		
Employment				9.821	0.007
Employed	1254 (28.7)	460 (28.9)	794 (28.6)		
Retired	902 (20.7)	290 (18.2)	612 (22.1)		
Unemployed	2210 (50.6)	842 (52.9)	1368 (49.3)		
Marital status ^a^				194.069	<0.001
Single	894 (20.5)	505 (31.7)	389 (14.0)		
Couple	3472 (79.5)	1087 (68.3)	2385 (86.0)		
Insurance ^b^				6.997	0.072
URRBMI	3449 (79.0)	1276(80.2)	2173 (78.3)		
UEBMI	799 (18.3)	265 (16.6)	534 (19.3)		
Others	49 (1.1)	23 (1.4)	26 (0.9)		
None	69 (1.6)	28 (1.8)	41 (1.5)		
Self-rated economic status ^c^				28.118	<0.001
Q1	943 (21.6)	323 (20.3)	620 (22.4)		
Q2	3056 (70.0)	1090 (68.5)	1966 (70.9)		
Q3	332 (7.6)	165 (10.4)	167 (6.0)		
Q4	35 (0.8)	14 (0.9)	21 (0.8)		
Self-rated health status				6.374	0.041
Good	1825 (41.8)	635 (39.9)	1190 (42.9)		
Medium	1456 (33.3)	529 (33.2)	927 (33.4)		
Bad	1085 (24.9)	428 (26.9)	657 (23.7)		
Chronic diseases				5.847	0.016
Yes	4058 (92.9)	1460 (91.7)	2598 (93.7)		
No	308 (7.1)	132 (8.3)	176 (6.3)		
Self-treatment				54.891	<0.001
Yes	3490 (79.9)	1178 (74.0)	2312 (83.3)		
No	876 (20.1)	414 (26.0)	462 (16.7)		

^a^ single: not married/divorced/widowed or others; ^b^ URRBMI: Urban and Rural Residents Basic Medical Insurance; UEBMI: Urban Employee Basic Medical Insurance; ^c^ self-rated economic status, quartile 1 is the richest and quartile 4 is the poorest.

**Table 2:** Association of self-treatment and living arrangement in Shandong, China.

Characteristics	Model 1	Model 2	Model 3
	OR (95% CI)	OR (95% CI)	OR (95% CI)
Empty nest			
Yes	1.0	1.0	1.0
No	1.759 (1.514, 2.043) **	1.773 (1.517, 2.073) **	1.749 (1.496, 2.045) **
Gender			
Female		1.0	1.0
Male		0.931 (0.788, 1.100)	0.920 (0.779, 1.085)
Age			
60–69		1.0	1.0
70–79		0.886 (0.752, 1.044)	0.873 (0.740, 1.029)
≥80		1.050 (0.763, 1.445)	1.058 (0.769, 1.457)
Education			
No schooling		1.0	1.0
Primary school		0.856 (0.709, 1.103)	0.868 (0.719, 1.048)
Junior school and above		0.724 (0.575, 0.911) *	0.721 (0.573, 0.908) *
Employment			
Employed		1.0	1.0
Retired		0.964 (0.707, 1.313)	0.944 (0.692, 1.288)
Unemployed		1.157 (0.961, 1.393)	1.141 (0.947, 1.375)
Marital status ^a^			
Couple		1.0	1.0
Single		1.169 (0.953, 1.433)	1.145 (0.935, 1.402)
Insurance ^b^			
URRBMI		1.0	1.0
UEBMI		0.958 (0.715, 1.282)	0.957 (0.714, 1.283)
Others		0.394 (0.208, 0.744) *	0.360 (0.191, 0.681) *
None		1.234 (0.613, 2.483)	1.210 (0.598, 2.448)
Self-rated economic status ^c^			
Q1		1.0	1.0
Q2		0.746 (0.604, 0.922) *	0.764 (0.618, 0.945) *
Q3		0.787 (0.559, 1.108)	0.790 (0.561, 1.113)
Q4		0.744 (0.326, 1.695)	0.719 (0.316, 1.636)
Self-rated health status			
Good			1.0
Medium			0.976 (0.816, 1.167)
Bad			0.816 (0.671, 0.991) *
Chronic diseases			
No			1.0
Yes			1.836 (1.411, 2.390) **

* Significant at *p* < 0.05; ** Significant at *p* < 0.001; OR: odds ratio; CI: confidence interval; ^a^ single: not married/divorced/widowed or others; ^b^ URRBMI: Urban and Rural Residents Basic Medical Insurance; UEBMI: Urban Employee Basic Medical Insurance; ^c^ self-rated economic status, quartile 1 is the richest and quartile 4 is the poorest.

**Table 3:** Factors with self-treatment among empty nesters in Shandong. China. (*n* = 1592).

Characteristics	Self-Treatment	COR (95%CI)	*p*	AOR (95%CI)	*p*
	Yes (%)				
Total	1178 (74.0)				
Gender					NA
Female	774 (75.5)	1.0			
Male	404 (71.3)	0.804 (0.638, 1.013)	0.064		
Age					
60–69	559 (73.9)	1.0		1.0	
70–79	499 (72.2)	0.916 (0.726, 1.156)	0.459	0.809 (0.635, 1.031)	0.087
≥80	120 (82.8)	1.692 (1.067, 2.681)	0.025	1.367 (0.851, 2.196)	0.196
Education					
No schooling	406 (77.9)	1.0		1.0	
Primary school	472 (73.0)	0.764 (0.583, 1.001)	0.051	0.743 (0.563, 0.981)	0.036
Junior school and above	300 (70.8)	0.685 (0.511, 0.920)	0.012	0.593 (0.423, 0.809)	0.001
Employment					NA
Employed	341 (74.1)	1.0			
Retired	215 (74.1)	1.000 (0.715, 1.399)	0.998		
Unemployed	622 (73.9)	0.987 (0.761, 1.279)	0.919		
Marital status ^a^					NA
Couple	791 (72.8)	1.0			
Single	387 (76.6)	1.227 (0.960, 1.569)	0.102		
Insurance ^b^					NA
URRBMI	945 (74.1)	1.0			
UEBMI	197 (74.3)	1.015 (0.750, 1.374)	0.925		
Others	14 (60.9)	0.537 (0.222, 1.297)	0.167		
None	22 (78.6)	1.266 (0.493, 3.253)	0.625		
Self-rated economic status ^c^					
Q1	264 (81.7)	1.0		1.0	
Q2	798 (73.2)	0.611 (0.447, 0.835)	0.002	0.588 (0.424, 0.815)	0.001
Q3	109 (66.1)	0.435 (0.283, 0.668)	< 0.001	0.487 (0.306, 0.776)	0.002
Q4	7 (50.0)	0.223 (0.076, 0.661)	0.007	0.297 (0.093, 0.952)	0.041
Self-rated health status					
Good	490 (77.2)	1.0		1.0	
Medium	392 (74.1)	0.847 (0.647, 1.108)	0.225	0.866 (0.658, 1.140)	0.306
Bad	296 (69.2)	0.664 (0.503, 0.875)	0.004	0.776 (0.577, 1.043)	0.093
Chronic diseases					
No	88 (66.7)	1.0		1.0	
Yes	1090 (74.7)	1.473 (1.007, 2.155)	0.046	1.548 (1.045, 2.294)	0.029

COR: crude OR; AOR: adjusted OR; NA: not applicable; ^a^ single: not married/divorced/widowed or others; ^b^ URRBMI: Urban and Rural Residents Basic Medical Insurance; UEBMI: Urban Employee Basic Medical Insurance; ^c^ self-rated economic status, quartile 1 is the richest and quartile 4 is the poorest.

**Table 4:** Factors with self-treatment among non-empty nesters in Shandong. China. (*n* = 2774).

Characteristics	Self-Treatment	COR (95%CI)	*p*	AOR (95%CI)	*p*
	Yes (%)				
Total	2312 (83.3)				
Gender					NA
Female	1471 (84.3)	1.0			
Male	841 (81.7)	0.826 (0.673, 1.013)	0.066		
Age					NA
60–69	1205 (83.2)	1.0			
70–79	930 (83.5)	1.023 (0.830, 1.262)	0.828		
≥80	177 (83.9)	1.054 (0.712, 1.560)	0.792		
Educational level					NA
No schooling	811 (84.6)	1.0			
Primary school	954 (83.7)	0.936 (0.740, 1.184)	0.582		
junior school and above	547 (81.0)	0.780 (0.601, 1.012)	0.061		
Employment					
Employed	638 (80.4)	1.0		1.0	
Retired	500 (81.7)	1.092 (0.834, 1.429)	0.524	1.190 (0.784, 1.808)	0.414
Unemployed	1174 (85.5)	1.480 (1.174, 1.866)	0.001	1.542 (1.208, 1.969)	0.001
Marital status ^a^					NA
Couple	1977 (82.9)	1.0			
Single	335 (86.1)	1.280 (0.942, 1.740)	0.114		
Insurance ^b^					
URRBMI	1818 (83.7)	1.0		1.0	
UEBMI	441 (82.6)	0.926 (0.720, 1.190)	0.548	0.894 (0.605, 1.321)	0.572
Others	17 (65.4)	0.398 (0.172, 0.921)	0.031	0.344 (0.145, 0.814)	0.015
None	36 (87.7)	1.518 (0.580, 3.973)	0.395	1.286 (0.479, 3.450)	0.618
Self-rated economic status ^c^					NA
Q1	515 (83.1)	1.0			
Q2	1631 (83.0)	0.993 (0.780, 1.263)	0.952		
Q3	147 (88.0)	1.499 (0.898, 2.501)	0.122		
Q4	19 (90.5)	1.937 (0.444, 8.441)	0.379		
Self-rated health status					NA
Good	989 (83.1)	1.0			
Medium	782 (84.4)	1.096 (0.868, 1.384)	0.441		
Bad	541 (82.3)	0.948 (0.737, 1.219)	0.676		
Chronic diseases					
No	127 (72.2)	1.0		1.0	
Yes	2185 (84.1)	2.041 (1.444, 2.885)	< 0.001	2.038 (1.436, 2.892)	<0.001

COR: crude OR; AOR: adjusted OR; NA: not applicable; ^a^ single: not married/divorced/widowed or others; ^b^ URRBMI: Urban and Rural Residents Basic Medical Insurance; UEBMI: Urban Employee Basic Medical Insurance; ^c^ self-rated economic status, quartile 1 is the richest and quartile 4 is the poorest.
